# Treatment of Acne

**Published:** 1882-08

**Authors:** 


### Treatment of Acne.

The treatment of Acne may be devided into two parts, the one as essential, perhaps, as the other. They are the constitutional and general and the local or special; and in each one of these, the greatest care on the part of the physician and patience on that of the patient are to be observed, as many good remedies may be fairly and conscientiously tried before an impression is made.

*Constitutional treatment* is to be directed on general principles and as good a therapeusis as possible should be carried out. Where there is a vitiated state of the system it ought to be corrected. Where constipation exists it should be carefully looked after. Saline laxatives should be given with an occasional small dose of calomel or blue mass, and mineral waters should be taken before dinner. Among these are the Ofner Racoczy, Friederichschall, Hunjadi Janos, and many others whose aperient properties are well-known. Duhring recommends the following:

| | | |
|---|---|---|
| ℞ | Magnesiæ Sulphatis, - - - | ℥jss. |
| | Ferri Sulphatis, - - - | gr xvj. |
| | Acidi Sulphurici dilut. - - - | ʒij. |
| | Aquæ destillati, - - - | ℥viij. |
| | M. Ft. Sol. | |

Sig. A tablespoonful in a goblet of water two or three times a day.

Should the patient be anæmic or scrofulous, cod-liver oil and good tonic treatment are indicated. Mineral acids especially are good. When iron is given, it should be done sparingly as it is reported by some to excite an eruption of acne; the same may be said of bromide of potassium which is sometimes given as an anaphrodisiac, and iodide of iron, as an antiscrofulous which when pushed, often produce the mischief it was extended to repair.

Arsenic was for a time supposed to exert almost a specific influence over acne; but, at present, it is not employed so extensively. It is found of service, at times, in the treatment of the indolent papular form or where the papules are imperfectly developed and is to be given as a tonic in doses of one to three drops of Fowler's solution two or three times a day after meals.

Gubler, of Paris, and Bulkley, of New York, have highly lauded the treatment of acne punctata and comedo by the internal administration of glycerine in tablespoonful doses three times a day. This method has been tried by others, but they deny its good effects, Taylor laying stress upon the fact of his failure to obtain any success.

Another remedy especially directed to this affection is one not long since re-introduced to the profession by Ringer and others. It is sulphide of calcium, which is to be taken in doses of one-tenth to one-half of a grain four times daily. Its chief value is in the suppurative form as it seems to have a tendency to arrest suppuration, making it a valuable remedy in furunculosis. It may do good in acne where there are large postules forming, but it should be carefully watched as W. T. Alexander has lately recorded cases in which the drug produced exactly the effect which it was intended to combat but as the author suggests it may merely be due to personal idiosyncrasy.

Diet and hygiene are two very important factors in the general treatment not only of acne, but of all diseases of the skin whether constitutional or local. Good air, good water, good food, exercise both mental and moral, should not be withheld.

Drs. Satterlee and Piffard, have employed electricity in acne. It would, I think, prove of service in those cases of apparent good health in which the eruption seems referable to some defect in nerve power or in the lessened nerve stimulation. The Faradic current is used, the positive pole being placed at the naps of the neck and the negative pole to the affected parts. This operation is to last some ten or fifteen minutes and is to be repeated every second or third day. There can be no doubt that this method is a good one as it stimulates the nerves of the skin and tends to bring about a normal state in regard to the functions of the sebaceous glands.

The importance of general treatment cannot be insisted upon too much as it plays a large part in the management and cure of that most troublesome and persistent affection—acne vulgaris.

*Local Treatment* is of the most varied character, being in the main either stimulating or soothing. The mainstay and sheet-anchor in acne is, beyond doubt, sulphur and its preparations. Erasmus Wilson is credited as being the first to propose sulphur lotion as an application in acne punctata and it is yet regarded as of the highest importance. It is the remedy which is, after all, the safest and most reliable. It is to be used in the form of an ointment or lotion in the proportion of one-half to one drachm to the ounce. The following are the formulæ of three excellent lotions, each one said to be reliable.

℞ Sulphuris loti, - - - - ʒi.

Ætheris - - - - ʒvj.

Alcoholis, - - - - ℥iijss.

M.

Sig. Apply as a lotion. (Bulkley.)

℞ Sulphuris precip.
Potassii bicarb.
Aquæ laurocerasi.
Alcoholis aa ℥j.
M.
Sig. Apply as a lotion. (Hebra.)

℞ Sulphuris precip, - - - - ʒij.
Pulv. Camphoræ, - - - gr. x.
Pulv. tragacanth, - - - - ℈j.
Aquæ calcis,
Aquæ rosæ, - - - - aa ℥ij.
M.
Sig. Use as a lotion. (Kummerfeld.)

Duhring recommends an unguent to be rubbed in at night which is also useful; it is as follows:

℞ Sulphuris precip, - - - - ʒi.

Glycerine puriss, - - - ʒss.

Adipis benz, - - - - ℥j.

Ol. rosæ. - - - - gtt. iij.

M.

James Gage Persons, recommends the use of precipitated sulphur in its dry state. It is to be applied to the face with an ordinary powder puff as face powder is, and, by the addition of a few drops of some essential oil, it makes a good cosmetic. He claims that this is the most rapid in its action. I have employed this method and am well pleased with it, both on account of its efficacy and the favor it finds with patients.

In many indolent cases a stimulating course of treatment will be necessary and washing with sapo viridis with thorough friction of the skin will be found of benefit. The following is also a good stimulant application : take of sapo viridis, alcohol and rose-water, each one part. If not sufficiently strong take of the soap two parts and of alcohol one.

In acne punctata, Hillairet recommends touching the points of the papules with tincture of iodine or of cantharides and as a milder stimulant washing with a solution containing water, 250 parts; borax, 10 to 15; and sulphuric ether 8 to 15. When the skin again becomes normal in its action modify the secretions by means of astringent lotions.

Sulphuret of potassium is a good local application in a strength of five to twenty-five grains to the ounce. It is particularly serviceable in the papular form. Prof. Hardy, of Paris, combines it as follows :

℞ Potassii Sulphuret,
Tinct. benzoin, - - - aa gr. viij.
Aquæ rosæ, - - - - ℥j.
M.
Sig. Apply night and morning.

Where very active stimulation is required the biniodide of mercury has also been highly spoken of in the strength of five to ten grains to the ounce. Corrosive sublimate is a great favorite with some but it is uncertain in its action. It is given in the strength of one-eighth to one grain to the ounce and its best vehicle is an emulsion of almonds.

Neumann recommends mercurial plaster applied on cloths, during the night, for severe cases of indurated acne.

Hutchinson, Morris, Liveing, and other English authors greatly recommend a method which consists in taking a glass rod, dipping it in a solution of acid nitrate of mercury and lightly touching the summits of the pustules, care being taken to use blotting paper to absorb the excess of remedy. No matter how carefully done, it may result in leaving scars much worse looking than the pustules, and, on that account, it is not in very great favor.

Besides, we have other local means which are more purely mechanical, or rather surgical in character. The glands should have their contents squeezed out in order to relieve their walls from pressure and the best method of doing this is by means of a comedone extractor.

In sluggish papules accompanied by comedones the dermal curette is of great value, as it brings on a healthy inflammation of the tissues to replace the chronic infiltration. In acute acne indurata scarifications should be made freely and hot water applied to promote the bleeding and relieve the engorgement. This establishes a tendency to resolution.

When the pustules are large, or when there is a suspicion of pus in a papule, the proper treatment is to cut them open so as to give free exit to the contained pus. If there be a superficial distension of the blood-vessels, it is not only proper but imperative to cut open the papules so as to relieve the vessels. The proper dressings are then to be applied. Where the papules remain miliary they should be opened with a milium needle.

You will find, no doubt, that I have omitted many remedies and methods, but the local measures, in this affection, are so numerous and varied that in would be well-nigh impossible for me to enumerate all of them. I have endeavored to give the principal ones.

The following are some of the substances used in the local treatment of acne: Carbolic acid, aconite, chloride of antimony, lemon juice, collodion, chloride of iron, peroxide of iron, cyanide of mercury, potassa, chlorate of potash, borax, talc, iodide of patassium, ammoniated mercury, albuminate of mercury, oil of cade, vaseline, chrysophanic and a host of others.

The treatment depends upon the observation of the physician, who must determine whether stimulating or soothing treatment is to be employed, and in either case he will only have *l'embarras de richesse.* The surgical methods to be used should never be forgotten, as they are by far the most satisfactory in their results.

